# Gastroenteritis Outbreaks after Contamination of Water Supply Systems: Public Health Response Gaps and Challenges, Greece, 2004–2023

**DOI:** 10.3390/ijerph21060701

**Published:** 2024-05-30

**Authors:** Theologia Sideroglou, Anthi Chrysostomou, Lida Politi, Leonidas Georgalis, Kassiani Mellou

**Affiliations:** 1 Department of Foodborne and Waterborne Diseases, Directorate of Surveillance and Prevention of Infectious Diseases, National Public Health Organization, 15123 Athens, Greece; t.sideroglou@eody.gov.gr (T.S.); a.chrysostomou@eody.gov.gr (A.C.); l.georgalis@eody.gov.gr (L.G.); 2 Department of Microbial Resistance and Infections in Health Care Settings, Directorate of Surveillance and Prevention of Infectious Diseases, National Public Health Organization, 15123 Athens, Greece; l.politi@eody.gov.gr; 3 Directorate of Surveillance and Prevention of Infectious Diseases, National Public Health Organization, 15123 Athens, Greece

**Keywords:** investigation, gastroenteritis outbreak, surveillance, waterborne, public health, Greece

## Abstract

Background: waterborne disease outbreaks (WGDOs) following the contamination of drinking water remain a public health concern. Methods: The current study aims to assess the occurrence and identify gaps in the notification and investigation of WGDOs in Greece. Data for 2004–2023 were retrieved and summarized. Results: Thirty-five outbreaks with 6128 recorded cases were identified. The median time from the date of onset in the first cases to reporting was 7 days (range: 1–26 days). Authorities were informed by health care services in thirty (85.7%) outbreaks and by the media in five (14.3%). The investigation methods used varied. An analytical study was conducted in nine (25.7%) outbreaks and the testing of clinical samples in twenty-seven (77.1%). In three (11.1%) outbreaks, clinical samples were simultaneously tested for multiple bacteria, viruses, and parasites. Water samples were collected in nineteen (54.3%) outbreaks (in three after chlorination) with a mean time lag of 5 days (range: 1–20 days) from the first cases. A pathogen in clinical samples was identified in 20 (57.1%) outbreaks and, in 1 (6.25%), the same microorganism was isolated in both clinical and water samples. Conclusions: delays in reporting and the heterogeneity of investigations depict that the surveillance of WGDOs and response practices should be strengthened, and operational procedures should be standardised.

## 1. Introduction

Safe drinking water is an essential for the quality of human life [1]. However, water quality can be compromised by its contamination with pathogenic microorganisms, which can cause illnesses in humans and onsets of outbreaks [2]. Waterborne disease outbreaks (WGDOs) following the contamination of drinking water remain a public health concern, not only in developing, but also in developed, countries [3,4].

It is difficult to measure the burden of WGDOs. It is estimated that globally one million deaths per year are attributable to unsafe water supply, sanitation, and hygiene [5]. In the World Health Organization European Region, approximately 18% of investigated outbreaks may be associated with water consumption [6]. According to the latest published data in 2022, 13 European Union Member States reported 41 WGDOs in total [7].

Several zoonotic agents, such as viruses, bacteria, and parasites, are often detected in the water, as well as in human samples in the context of WGDOs. *Cryptosporidium parvum*, norovirus, *Giardia lamblia*, *Campylobacter* spp. and rotavirus are more frequently implicated in large WGDOs [1,8]. Animal and human faeces constitute the most common source of contamination of water supplies [9], resulting in WGDOs of mixed aetiology, the frequency of which has increased in recent decades [10,11]. This was probably due to the improvement of available laboratory methods, but also due to changes in livestock management and climate change effects, such as extreme rainfall events and prolonged water scarcity [12,13,14].

The aim of this study is to assess the occurrence of WGDOs during 2004–2023, identify gaps in their notification and investigation, and depict the need for the implementation of a national management plan and standardised operating procedures (SOPs) for mitigating the risk of waterborne diarrhoeal diseases.

## 2. Methods

### 2.1. Definitions

A waterborne disease outbreak was defined as an incident in which two or more epidemiologically linked persons (cases) exhibited similar illness after exposure to the same water source, and in which epidemiological data implicated the water as the likely source of the illness [15]. Exposure to a water source was defined as the consumption of drinking water of the same source. Drinking water refers to water intended for human consumption and includes water collected, treated, stored, or distributed in public and individual water systems [16]. Legionellosis outbreaks and outbreaks caused by water not intended for human consumption were excluded from the analysis.

### 2.2. Data Source

In Greece, the surveillance of WGDOs was introduced in the Mandatory Notification System (MNS) of the Hellenic National Public Health Organization (HNPHO) in 2004, in accordance with the European Union surveillance framework. Surveillance is mainly passive. Physicians and microbiologists working in the public or private sector are requested to report clusters of cases with gastrointestinal symptoms to the local public health authorities and the HNPHO. Laboratory confirmation of the aetiological agent is not a prerequisite for reporting.

The reporting of cases is achieved by filling in a specific notification form. This notification form includes aggregated data on the size (number of cases) and the burden (number of hospitalised patients and deaths) of the outbreak. It also includes information on the place (place of residence or exposure) and time (date of symptoms’ onset), as well as the available demographic (sex and age) and the clinical and laboratory characteristics of the cases.

Apart from the MNS, information on WGDOs may derive from event-based surveillance systems. Once a signal is verified, a risk assessment of the event takes place and response mechanisms are triggered.

The epidemiological investigation of WGDOs is conducted by the HNPHO and the local public health directorates. Information on signals of potential events and the results of the epidemiological, laboratory, and environmental investigations are recorded in an integrated database and, in compliance with the Centres for Disease Control and Prevention guidelines, they are classified in the four classes based on the available level of evidence. Specifically, an outbreak is classified as class I when epidemiological data [the relative risk (RR) or odds ratio (OR) linking outbreak cases to the same water exposure is equal to or higher than two (≥2) and the *p*-value is equal to or less than 0.05 (≤0.05)], alongside supporting available laboratory data (e.g., water quality, environmental, or microbiological), are available. When epidemiological data, without supporting laboratory data, are available, then the outbreak is categorized as class II. In the case of the availability of laboratory data (e.g., water quality, environmental, or microbiological) alone, then the outbreak is classified as class III. Finally, when neither epidemiological nor laboratory data are available, then the outbreak is classified as class IV [17].

### 2.3. Statistical Analysis

Data on recorded WGDOs during 2004–2023 were extracted from the abovementioned integrated database. The proportion of outbreaks notified by medical services was calculated, as well as the delay in reporting (time lag between the onset of symptoms of the first recorded case and the outbreak notification). An interrupted time series analysis was performed on the annual number of reported outbreaks from 2004 to 2023 to assess temporal trends (a negative binominal regression model). To assess the geographical distribution of WGDOs, the mean annual notification rate per 1,000,000 people was calculated by region. Proportions of conducted investigations were calculated, and the results of epidemiological, laboratory, and environmental investigation were summarized. An analysis of the data was carried out with STATA version 16 software (Stata Corporation LP, College Station, TX, USA). All statistical tests were two-tailed.

### 2.4. Ethical Considerations

Already-collected data were anonymised and sent to the HNPHO, the competent authority for the surveillance of communicable diseases under Greek law. Onwards, data were entered in the integrated database at the HNPHO’s premises, processed, and analysed according to national and European Union laws. Furthermore, in terms of publication in a scientific journal, a data request was submitted to the Scientific Board of EODY (submission code and date: 6517/01 April 2024, acceptance code and date: 8819/13 May 2024).

## 3. Results

### 3.1. Notification of WGDOs, 2004–2023

During the period of 2004–2023, 43 community WGDOs were recorded. In total, 8 outbreaks, with 340 cases in total, which were attributed to the consumption of water not intended for human consumption, originating from wells (*n* = 3) or rivers (*n* = 5), were excluded from the analysis. Overall, 35 outbreaks related to the public water supply system were analysed. The median annual number of reported outbreaks was two (2), ranging from one to four outbreaks per year. In time-series analysis, there was no statistically significant decreasing or increasing trend in the number of identified outbreaks from 2004 to 2023 (*p* = 0.984).

Twenty-one (60.0%) outbreaks were reported through the MNS, while the initial signal about the increased number of gastroenteritis cases came through the media for five (14.3%) outbreaks. Finally, for nine (25.7%) outbreaks, the staff of health care units actively called the HNPHO to report the increase in gastroenteritis cases.

### 3.2. Timeliness of Reporting

The outbreaks were reported with a median delay of 7 days (range: 1–26 days) from the onset of symptoms of the first recognized case. For 30 outbreaks, with the respective information available, 3 (10%) were notified during the first 24 h of the symptoms’ onset in the first case, 6 (20%) during the 48 h, 6 (20%) outbreaks in the period of >48 h—days, and 15 (50%) in more than 7 days from the date of symptoms’ onset in the first case. 

Six (17.1%) outbreaks were reported after the date of symptom’s onset of the last recorded case.

### 3.3. Time and Place of the Outbreaks

**Figure 1:**
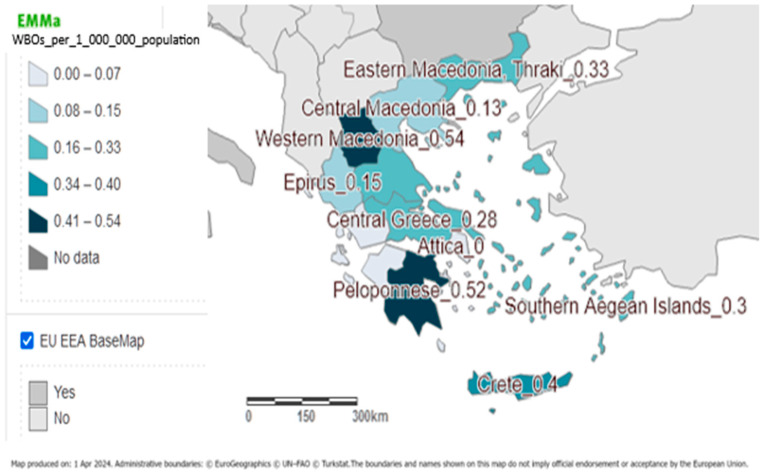
Mean annual notification rate (outbreaks/1,000,000 people) of gastroenteritis waterborne disease outbreaks by region, Greece, 2004–2023.

WGDOs were reported in 11 (84.6%) of the 13 regions of the country. In Attica and Ionian islands, no WBOs were reported. The highest mean annual notification rate for the period of 2004–2023 was recorded for the region of Western Macedonia (0.54 outbreaks/1,000,000 population). The mean annual notification rate of reported outbreaks by region for the period 2004–2023 is depicted in Figure 1.

**Figure 2:**
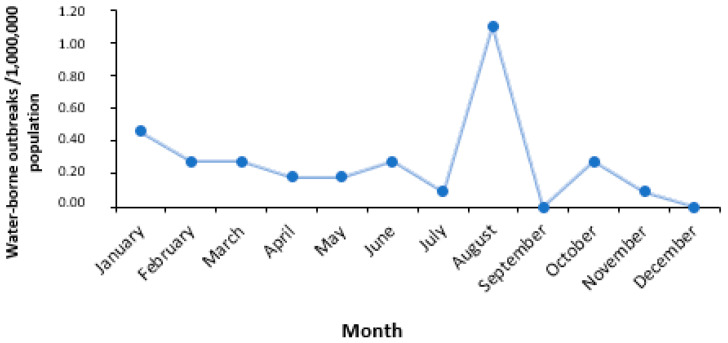
Mean monthly notification rate of gastroenteritis waterborne outbreaks (number of outbreaks/1,000,000 people), Greece, 2004–2023.

Twelve outbreaks (34.3%) occurred in August. The mean monthly notification rate of outbreaks per 1,000,000 people is depicted in Figure 2.

### 3.4. Size of the Outbreaks

A total number of 6128 cases was recorded, with a median of 54 cases (range: 2–1640) per outbreak. Thirty-three (94.3%) outbreaks involved cases from more than one household, and thirty-two (91.4%) had more than ten cases each. Seven outbreaks with more than two hundred cases were recorded. The total number of cases in those 7 outbreaks accounted for 76.5% of total cases (4686 out of the 6128 cases). Overall, the outbreaks had a median duration of 10 days (range: 1–45 days).

### 3.5. Demographics and Severity of Disease

People of all genders and age groups were equally affected (*p* > 0.05). From the 3678 (60.0%) cases, according to available information, 218 (5.9%) were hospitalised. No deaths were recorded.

### 3.6. Analytical Epidemiology

In the analytical studies conducted in nine outbreaks, the estimated value of the measure of association (odds ratio or relative risk) was higher than two, and the analysis resulted in a statistically significant association between the consumption of tap water and the occurrence of gastroenteritis symptoms. Regarding the study design, in six (66.7%) outbreaks, a case–control study was conducted. In one (11.1%), a cohort study was performed, whereas in one (11.1%) outbreak, both study designs were implemented. On one (11.1%) occasion, a case–control and a case-crossover study were performed. The characteristics of the outbreaks investigated with an analytical study are summarized in Table 1.

### 3.7. Laboratory Investigation of Clinical Samples

A laboratory investigation of clinical samples was carried out in 27 (77.1%) of the 35 WGDOs. In 16 (59.3%) outbreaks, clinical samples were tested for *Salmonella* spp. and *Shigella* spp. at the local hospitals of the affected areas, while testing for viruses (norovirus and rotavirus) took place in 11 (40%) outbreaks. Out of 11 outbreaks in which testing for viruses was performed, in 8 (72.7%), testing was conducted in the public health laboratories network of the HNPHO. Testing for parasites was not reported. Laboratory testing was based on the available resources, as well as on the clinical manifestations of cases. The median number of tested samples by outbreak was 7 (range: 1–35 samples). An aetiological agent was identified in 20 (57.1%) WGDOs. The distribution of WGDOs by identified causative agents is given in Table 2. Norovirus was the most frequently identified pathogen; it was detected in clinical samples in seven (35%) out of the twenty outbreaks.

In three outbreaks that occurred and were investigated in 2019, 2020, and 2022, clinical samples were tested with multiplex polymerase chain reactions (multiplex-PCR) for the simultaneous detection of 22 pathogens (Enterotoxigenic *E. coli* (ETEC), Enteropathogenic *E. coli* (EPEC), Enteroaggregative *E. coli* (EAEC), and Shiga-like toxin-producing *E. coli* (*stx1 and stx2*), with the specific detection of *E. coli* O157, *Shigella/Enteroinvasive E. coli* (*EIEC*), *Campylobacter* (*jejuni/coli/upsaliensis*), *Vibrio* (*parahaemolyticus/vulnificus/cholerae*), *Yersinia enterocolitica*, *Plesiomonas shigelloides*, *Clostridium difficile* (Toxin A/B), *Salmonella* spp., *Cryptosporidium* spp., *Cyclospora cayetanensis*, *Entamoeba histolytica*, *Giardia lamblia*, Adenovirus F40/41, Astrovirus, norovirus GI/GII, rotavirus A, and Sapovirus (genogroups I, II, IV, and V), using the BioFire FilmArray gastrointestinal panel [18]. Based on the results of the multiplex PCR, two out of the three outbreaks were of mixed origin.

### 3.8. Environmental Investigation

Environmental investigation was performed in all 35 WBOs. Water samples were collected and tested for bacterial indicators of faecal contamination in 19 (54.3%) WBOs. The median number of water samples tested by outbreak was 5 (range: 2–23 samples). Water sampling was performed with a mean delay of 5 days (range: 1–20) after symptoms’ onset in the first cases. Water sampling after the chlorination of the water supply system was reported in three (20%) outbreaks (Table 1). The causative agent was isolated in both clinical and water samples in one (6.25%) of the sixteen WBOs, for which the respective samples were collected [19].

Thirteen outbreaks (37.1%) were attributed to water source contamination, twelve (34.3%) to failures of the chlorination system, and five (14.3%) to the contamination of the water distribution system after damage to the pipelines. In five (14.3%) outbreaks, no factors contributing to the occurrence of the outbreak were identified.

In all outbreaks, recommendations on hygiene measures were given. In 11 (50.0%) outbreaks, the public health measures included recommendations regarding the safety of water consumption from the public water supply system, such as advice on the use of bottled water or boiling water before consumption. Finally, in 11 (50%) outbreaks, hyperchlorination (a temporal increase in the free chlorine residual in the water distribution system) was applied [20].

### 3.9. Classification of WGDOs

Eight (22.9%) out of the thirty-five WBOs were classified as class I, indicating that adequate epidemiological and water quality data were provided for the waterborne origin of the outbreak, according to CDC’s classification. One (2.9%) was classified as class II, thirteen (37.1%) as class III, and thirteen (37.1%) as class IV.

## 4. Discussion

In total, we studied 35 WBOs notified in Greece during 2004–2023. The number of reported outbreaks was stable over the years according to our analysis, and the notification rate was rather low, which was in accordance with that of other European countries [7]. However, the actual number of WGDOs during the study period remains unknown, as the identification of such outbreaks is challenging, leading to possible under-reporting, and therefore numbers could have been underestimated. Waterborne pathogens can also be spread in other ways (through food or via person-to-person or animal-to-person transmission), and linking illness to drinking water can be difficult, as most people are exposed to this possible risk factor [21]. For this reason, the performance of analytical epidemiological studies and laboratory investigations of clinical and water samples are of great importance.

The total number of outbreak-related cases in the 35 recorded WGDOs was high, which is a constant finding in the literature [22]. Identified outbreaks were attributed to the consumption of water from the public water supply system. This is an expected finding, given that in Greece drinking water is provided through public supply systems and only rarely does it come from private networks [23]. This could explain the difference observed in reports from other countries regarding the type of water supply in WGDOs [9].

A clear geographical pattern was not identified. It should be mentioned that in the region of Attica, which has 3,814,064 permanent residents [24], no WGDOs were reported. However, outbreaks occurred in almost all other regions of the country, indicating that a WBO could occur anywhere in the country. The prevailing pattern of outbursts of WGDOs during the summer period may be a result of extreme temperatures. These facilitate pathogen propagation and may lead to water scarcity and reduced water quality due to improper water transport and storage in water tanks in remote areas (such as islands). Also, increased population density in touristic areas may generate further stress on available water sources and sanitation systems [25].

Delays in notification are an important limitation in the investigation of WGDOs. Even though the surveillance of WGDOs is included in the MNS, health care services do not always report the observed increased number of gastroenteritis cases, and initial information comes through other sources with delay. The lack of an efficient designated surveillance system for the detection of WGDOs and delays in reporting have also been reported by other countries [26,27]. Efforts to increase awareness should be made, and educational workshops for physicians need to be organized regularly to tackle delays in the notification chain. This could ultimately lead to a desired increase in timeliness and the consequent prevention of the widespread exposure of the population [28,29]. Moreover, the enhancement of event-based surveillance systems should become a priority in order to capture sudden increases in gastroenteritis cases, especially of those with mild clinical manifestations, since the MNS is not fit for this purpose.

Regarding investigations, an analytical epidemiological study was not conducted for all large outbreaks. This finding was also demonstrated by the results of the CDC’s classification. The decision to perform analytical studies was not made according to a standard set of criteria (the number of cases, severity of clinical manifestations, etc.). The introduction of such criteria is critical. The added value of analytical epidemiology is of great importance, as it provides evidence on the waterborne origin of the outbreak even when the laboratory confirmation of the contamination of water is not possible.

Additionally, even though *Salmonella* spp. and norovirus were the most frequently identified causative agents, in accordance with the findings from other European countries [9], laboratory investigations did not lead to the identification of a pathogen in almost half of the reported outbreaks. Testing was not homogenous and was not conducted for a standard list of pathogens in every outbreak. This was mainly due to the lack of capacity for the testing of clinical samples at the local level, especially for viruses and parasites, while no specific protocol was followed regarding the number of clinical specimens to be tested in each outbreak. Given that the probability of a mixed aetiology of WGDOs is high, the testing of clinical samples should consider a wide spectrum of pathogens. Recent practices include the use of molecular methods for the simultaneous detection of multiple pathogens in a single test, making the laboratory investigation of clinical samples more efficient. These techniques should be included in an integrated response protocol for WGDOs.

A main challenge during the conducted outbreak investigations was also the delay of water sampling, or the sampling of water after the chlorination of the system. No specific protocol was followed, and testing was mostly restricted to specific microbiological indicators according to the legislation [11,30]. Although testing for faecal-indicator bacteria is crucial, laboratory investigation focusing on specific pathogens is very important for the identification of the causative agent of the outbreak. A need for the enhancement of the capacity and financing of the local laboratories for testing a wide spectrum of pathogens in water samples is evident.

In parallel with the difficulty in confirming the water source, the route of contamination of the water supply system and the factors that contributed to the contamination remained unknown in several cases. As the majority of WGDOs have been reported in remote areas, outbreak investigations demonstrate the importance of timely notification cases and of the development of SOPs for water sampling [11,31]. The inability to find the factors that contributed to the contamination of the system can also be attributed to the lack of water safety plans, which is the result of gaps in legislation and in policy frameworks and resources, both financial and human. Finally, the lack of action plans, preparedness protocols, and training of the local authorities to conduct environmental investigations in the context of an outbreak, despite the fact that the World Health Organization has stressed their importance, highlight the response limitations [32]. This became evident in the case of a *S.* Bovismorbificans outbreak [19], where the pathogen was identified in both clinical and water samples. Although the coordination of the investigation was initiated promptly, the notification itself was delayed. This led to further detained environmental investigations, and ultimately to the failure to reveal the way the water might have been contaminated with *Salmonella* spp.

Our study had some limitations. The data were collected and reported to the HNPHO by healthcare professionals at the local level. In many instances, the reporting of an outbreak was conducted after the event was over, and data were incomplete. The delays made active case finding and epidemiological investigations time-consuming and may have led to the failure of identifying the origin and route of contamination. In many cases, by the time HNPHO was notified of an outbreak, measures had already been taken at the local level, thus making water sampling unrepresentative. Additionally, faecal samples were not always collected from patients. This was either due to a lack of laboratory capacity at the local level, or because the symptoms had ceased by the time the patients sought medical assistance. These reasons led to an inadequate laboratory investigation of clinical samples. Moreover, for several outbreaks, many patients exhibiting symptoms may have chosen to use over-the-counter medication and not visit a healthcare facility. Therefore, under-ascertainment and under-reporting in several cases led to small sample sizes in the analytical studies conducted. Despite the small sample sizes in outbreaks where an analytical study was conducted, a statistically significant association between the consumption of water from the public system and illness was revealed. This underscores the importance of conducting such studies and following international guides and protocols for waterborne disease outbreak investigations [33]. Finally, the lack of specific criteria and standardised communications protocols did not allow for the performance of an analytical epidemiological investigation for every community outbreak.

## 5. Conclusions

Our study reveals that WGDOs remain a public health concern in Greece, as in other European countries. Delays in reporting and the lack of a harmonised approach for the WGDO investigations underline the need for and the high importance of available integrated response protocols that can be followed according to specific criteria. Available international toolkits and protocols on waterborne outbreak investigations specify the need for epidemiological investigations—descriptive and analytical studies—and laboratory investigations of both clinical and environmental samples, along with the inspection of facilities from the source to the end point of the public water system. Therefore, protocols should focus on the early detection and notification of such outbreaks, on in-depth epidemiological and environmental investigations, on the optimal laboratory testing of clinical and water samples, as well as on optimal coordination and communication between health authorities, water supply companies, and the HNPHO. The failure to identify where exactly the contamination occurred deprives us of evidence on the specific weak points of the systems. However, it is documented that there is a lack of a national plan. SOPs, operations, and water supply system maintenance are mostly in the hands of local authorities and differ throughout the country. No specific requirements are in place for monitoring the water quality and the maintenance of the systems, which in most rural areas of the country are aged. Over recent decades, drinking water management policies have resulted in decreased disease burdens. However, related policies should be strengthened to address the remaining gaps.

## Figures and Tables

**Table 1:** Characteristics of waterborne disease outbreaks investigated with analytical study (*n* = 9), Greece, 2004–2023.

Time *	Region	Number of Cases	Number of Laboratory-Confirmed Cases	Type of Study	Measure of Association	Pathogen (Clinical Samples)	Water Sampling before or after Chlorination	Contributing Factors
15 February 2004–10 March 2004	Crete	37	35	CCS	OR = 10.91 (95%CI: 1.86–63.8)	*Salmonella typhimurium*	Unknown	- Water source contamination - Shortcomings in water sanitation procedures
27 May 2009–7 June 2009	Crete	54	54	CCS and CCrS	OR = 5.88 (95%CI: 1.75–20.0) OR = 4.39 (95%CI: 1.30–14.8)	*Campylobacter* *jejuni*	After	Shortcomings in water sanitation procedures
10 January 2012–16 February 2012	Central Macedonia	79	7	CS	RR = 2.34 (95%CI: 1.55–3.53)	Norovirus and Adenovirus	Before	- Water source contamination - Shortcomings in water sanitation procedures
6 March 2012–21 March 2012	Thessaly	552	38	CCS	OR = 2.18 (95%CI: 1.11–4.28)	Rotavirus	Before	- Water source contamination - Shortcomings in water sanitation procedures - Contamination of water distribution system
9 August 2015–30 August 2015	Central Macedonia	230	7	CCS	OR = 36.9 (*p* = 0.018)	Norovirus	Before	- Pipeline breakage - Contamination of water distribution system
25 January 2019–4 February 2019	Western Macedonia	638	11	CCS and CS	OR = 10 (95%CI: 2.09–93.4) RR = 2.22 (95%CI: 1.42–3.46)	Mixed aetiology (norovirus, *Campylobacter jejuni*, EHEC and EPEC)	After	Unknown
29 May 2020–18 June 2020	Peloponnese	87	6	CCS	OR = 10.9 (95%CI: 3.1–38.0)	Mixed aetiology (STEC, EPEC, *E. coli* O157)	Unknown	Shortcomings in water sanitation procedures
10 August 2022–24 August 2022	Peloponnese	33	15	CCS	OR = 5.46 (95%CI: 1.02–53.95)	*Salmonella* Bovismorbificans	Unknown	Unknown
2 March 2023–14 March 2023	Central Greece	39	1	CCS	OR = 3.02, (95%CI: 0.88–10.82)	Norovirus	Unknown	Unknown

* Time interval between the notification of the first and the last case recorded. OR = odds ratio; RR = relative risk; CCS: case–control study; CS: cohort study; CCrs: case-crossover study; EHEC: enterohemorrhagic *Escherichia coli*, EPEC: enteropathogenic *E. coli.*

**Table 2:** Distribution of notified waterborne disease outbreaks with an identified pathogen by causative agent (*n* = 20), Greece, 2004–2023.

Pathogen	*n* (%)
Norovirus	7 (35.0%)
*Salmonella* spp.	2 (10.0%)
*S.* Typhimurium	1 (5.0%)
*S*. Enteritidis	1 (5.0%)
*S.* Bovismorbificans	1 (5.0%)
*Shigella flexneri*	2 (10.0%)
*Campylobacter jejuni*	2 (10.0%)
Rotavirus	2 (10.0%)
Mixed aetiology	2 (10.0%)
Total	20 (100.0%)

## Data Availability

Data are unavailable due to privacy and ethical restrictions.
